# Expansion of vomeronasal receptor genes (*OlfC*) in the evolution of fright reaction in Ostariophysan fishes

**DOI:** 10.1038/s42003-019-0479-2

**Published:** 2019-06-21

**Authors:** Liandong Yang, Haifeng Jiang, Ying Wang, Yi Lei, Juan Chen, Ning Sun, Wenqi Lv, Cheng Wang, Thomas J. Near, Shunping He

**Affiliations:** 1 The Key Laboratory of Aquatic Biodiversity and Conservation of Chinese Academy of Sciences, Institute of Hydrobiology, Chinese Academy of Sciences, Wuhan, Hubei 430072 People’s Republic of China; 2 University of Chinese Academy of Sciences, 100049 Beijing, People’s Republic of China; 3 School of Life Sciences, Jianghan University, 430056 Wuhan, People’s Republic of China; 4 Department of Ecology and Evolutionary Biology and Peabody Museum of Natural History, Yale University, New Haven, CT 06520 USA; 5 Center for Excellence in Animal Evolution and Genetics, Chinese Academy of Sciences, 650223 Kunming, People’s Republic of China

**Keywords:** Evolution, Genetics

## Abstract

Ostariophysans are the most diverse group of freshwater fishes and feature a pheromone-elicited fright reaction. However, the genetic basis of fright reaction is unclear. Here, we compared vomeronasal type 2 receptor-like (*OlfC*) genes from fishes having and lacking fright reaction, to provide insight into evolution of pheromonal olfaction in fishes. We found *OlfC* genes expanded remarkably in ostariophysans having fright reaction compared with fishes lacking fright reaction. Phylogenetic analysis indicates *OlfC* subfamily 9 expanded specifically in ostariophysans having fright reaction. Principle component and phylogenetic logistic regression analysis partitioned fishes by ecotype (having or lacking fright reaction) and identified *OlfC* subfamily 9 as being an important factor for fright reaction. Expression levels of expanded *OlfC* subfamily genes after fright reaction in zebrafish changed more than did genes that had not expanded. Furthermore, evidence of positive selection was found in the expanded OlfC proteins in ostariophysan fishes having fright reaction. These results provide new insight into the genetic basis of fright reaction in ostariophysan fish and will enable future research into the mechanism of action of OlfC proteins.

## Abstract (web summary)

Yang et al. investigate the genetic basis of pheromone-elicited fright behavior in ostariophysan fish. They identify a subfamily of vomeronasal type 2 receptor-like (OlfC) that appears to be under strong positive selection; its transcriptional variation suggests a role in the fright behavior of ostariophysan fish.

## Introduction

Ostariophysan fishes are the largest and most diverse group of primarily freshwater fishes, representing about 28% of all known fish species and 68% of the world’s freshwater fishes^1^. The enormous ecological and evolutionary diversity of this group as well as the restricted distribution of almost all members to freshwater habitats has made this group a focus of research in evolutionary biology^2–6^. Previous studies have suggested that the common ancestor of the ostariophysan fishes entered freshwater about 251 million years ago, which coincides with the global decrease in oxygen levels in marine waters caused by the large mass extinction event that occurred at the end of the Permian era^4^. However, fishes invading freshwater habitats are expected to have faced stronger challenges to survive compared with those remaining in seawater habitats due to the presence of a different set of predators and the greater probability of encountering predators in the smaller freshwater environments. Therefore, ostariophysan fishes must have developed a set of mechanisms to adapt to the challenging, but promising, freshwater environment.

Among the mechanisms developed, the most remarkable one is the fright reaction that is found in almost all ostariophysan fishes^7–9^. The fright reaction is elicited by an alarm substance, which is a pheromone that is similar or identical in all ostariophysan fishes^1^. When a predator damages the skin of an ostariophysan fish, even with a minor injury, an alarm substance produced by epidermal club cells is released into the surrounding water. Nearby members of the same species, or sometimes closely related species, detect this waterborne alarm substance by smell, not taste, resulting in a species-specific fright reaction, which is assumed to be a defensive behavior against predators^8–10^. Thus, the fright reaction has been suggested to have made a marked contribution to the biological success of the ostariophysan fishes^11^; however, the genetic basis underlying fright reaction in ostariophysan fishes is still unclear.

Olfaction plays a crucial role in the daily life of fishes, including kin recognition, reproduction, and aggression^12^. Compared to the two distinct olfactory organs (the main olfactory epithelium and the vomeronasal organ) found in mammals^13^, fishes only have the main olfactory organ in each nasal cavity, the olfactory rosette^14^. Thus, all olfaction-related receptor genes are expressed in the olfactory epithelium of the nasal cavity in fishes. The main olfactory epithelium and the vomeronasal organ in mammals employ distinct receptors and signal transduction pathways, and excite different regions of the brain to mediate olfaction^13^. In mammals, the main olfactory epithelium mainly detects volatile odorants while the vomeronasal organ detects pheromones^15,16^, although there is some functional overlap between the main olfactory epithelium and the vomeronasal organ^17–21^.

In mammals, vomeronasal receptors (VNRs) are specifically expressed in the vomeronasal organ and are believed to encode receptors binding pheromone, which is a secreted or excreted chemical factor triggering a social response in members of the same species^22,23^. The mammalian VNR family is subdivided into two evolutionarily unrelated superfamilies: the VNR family 1 (V1R) and VNR family 2 (V2R)^24^. It has been suggested that V1R recognize small airborne pheromones^25^, whereas V2R bind to water-soluble pheromones^26^. Recently, it has been proposed that fish V1R-like and V2R-like receptors be named *ora* (olfactory receptor (OR) class A-related)^27^ and *OlfC* (OR class C-related)^28^, respectively. As fish do not have a vomeronasal system, the corresponding VNRs are expressed in the olfactory epithelium of the nasal cavity^14^. Previous studies have shown that there are enormous variations in the sizes of the V1R and V2R repertoires among different species and much of this variation can be explained as adaptation by the organisms to their different environments^29–31^.

Since the hypothesis that fright reaction plays crucial roles in the diversification of ostariophysan fishes, is elicited by pheromones, and fish *OlfC* genes recognize pheromones dissolved in water, we hypothesized that *OlfC* receptor genes in ostariophysan fishes have important roles in the fright reaction. Notably, some ostariophysan fishes lack the fright reaction, such as cave fish (*Astyanax mexicanus*) and electric eel (*Electrophorus electricus*)^32^. However, ostariophysan fishes lacking the fright reaction differ markedly in their way of life compared with ostariophysan fishes with the fright reaction. For example, ostariophysan fishes without the fright reaction typically are either cave dwelling, predaceous, nocturnal, electric, armored or solitary, or occupy cryptic habitats^11^, which suggests that their habitats reduce the need for this defense against predation. These species, therefore, provide an opportunity to test the association between *OlfC* receptor genes and the fright reaction in ostariophysan fishes. To examine the contributions of *OlfC* genes to the fright reaction in ostariophysan fishes, we have systematically defined the *OlfC* gene repertoires among ostariophysan fishes, both those having and lacking a fright reaction, and compared their complexity and evolution among the different groups of fishes. Our results showed that subfamily 9 of *OlfC* genes expanded substantially in ostariophysan fishes having the fright reaction, and their sequences show evidence of positive selection. The levels of gene expression in this expanded subfamily are elevated after stimulation with damaged skin from zebrafish. Our findings provide insights into the genetic basis of the fright reaction in ostariophysan fishes.

## Results

### Expansion of the number of *OlfC* genes in ostariophysans

**Fig. 1:**
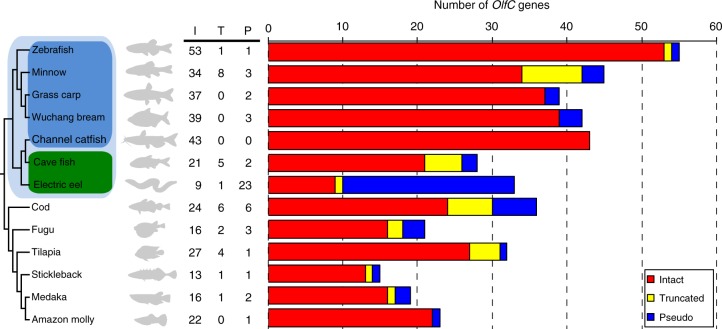
Numbers of *OlfC* genes in the genome sequences of 13 species of fish. “I,” “T,” and “P” indicate the numbers of intact genes, truncated genes, and pseudogenes, respectively. Branch shades represent the different groups of fishes: ostariophysan fishes (light blue); ostariophysan fishes having the fright reaction (deep blue); ostariophysan fishes lacking the fright reaction (deep green). Species tree was taken from previous studies^2,4,50^

We examined the genome sequences of a total of 13 species of fish, which were divided into three groups. The first group comprised five ostariophysan fishes having the fright reaction: zebrafish, minnow, grass carp, Wuchang bream, and channel catfish. The second group comprised two ostariophysan fishes lacking the fright reaction: cave fish and electric eel. Finally, the third group was made up of six non-ostariophysan fishes: cod, fugu, tilapia, stickleback, medaka, and Amazon molly. In all, 354 *OlfC* genes were identified in the genomes of these 13 fish species (Fig. 1). Among the identified *OlfC* genes, those from the zebrafish, fugu, stickleback, and medaka are updates from previous studies^33–35^, while those from the minnow, grass carp, Wuchang bream, channel catfish, cave fish, electric eel, cod, tilapia, and Amazon molly are newly identified in this study. First, we compared the results from our pipeline with those from previous reports. We found that the *OlfC* gene counts were similar between our results and previous reports (Supplementary Table 1 and Fig. 1), suggesting that our pipeline was robust and the number of identified *OlfC* genes are reliable. We then classified the identified *OlfC* gene into three categories: intact genes (with an intact open reading frame (ORF) and complete coding region), truncated genes (with an intact ORF but partial coding region), and pseudogenes (with disrupted ORF due to nonsense or frameshift mutations in the coding region). Intact and truncated genes are possible functional genes, whereas the pseudogenes are putative nonfunctional genes. For all of the species we examined, the number of truncated genes is generally small (Fig. 1); thus, the number of intact *OlfC* genes is likely a good indicator of the number of functional *OlfC* genes in each species. The deduced protein sequences of the identified intact *OlfC* genes are provided in Supplementary Data 1.

The total number of *OlfC* genes varies substantially among species (Fig. 1). Remarkably, the number of intact *OlfC* genes in ostariophysan fishes having the fright reaction (gene number ranges from 34 to 53) is almost twice that in ostariophysan fishes lacking the fright reaction (gene number is 9 and 21) and non-ostariophysan fishes (gene number ranges from 13 to 27). *OlfC* genes make up a statistically significantly higher proportion of the number of genes in the zebrafish than the cave fish genome (53/25465 = 0.21% vs. 21/23042 = 0.09%, *χ*^2^ test, *P* = 0.0009). To examine whether the observed increased size of the *OlfC* gene family in zebrafish was due to a specific increase in the size of this gene family or was part of a more general increase in sizes of gene families in zebrafish, compared with cave fish, we performed a genome-wide comparison of gene family sizes between these two species, based on the gene family annotations in Ensembl 83. We identified a total of 7360 gene families being shared between zebrafish and cave fish, with 3932 gene families having at least 2 genes in either zebrafish or cave fish retained for the subsequent analysis. We found that the ratio of the *OlfC* gene family repertoire size between zebrafish and cave fish of 2.52 (53/21) to be significantly higher than the mean ratio value of 1.16 for 3932 gene family sizes between the two genomes (one-sample Student’s *t* test, *P* *<* 2.2 × 10^−16^) (Supplementary Fig. 2A). When only larger gene families were used, qualitatively similar results were obtained (Supplementary Fig. 2B–D). These data suggest that the ostariophysan fishes having a fright reaction have significantly expanded the repertories of their *OlfC* gene family.

### *OlfC* subfamily 9 expanded exclusively in ostariophysans

**Fig. 2:**
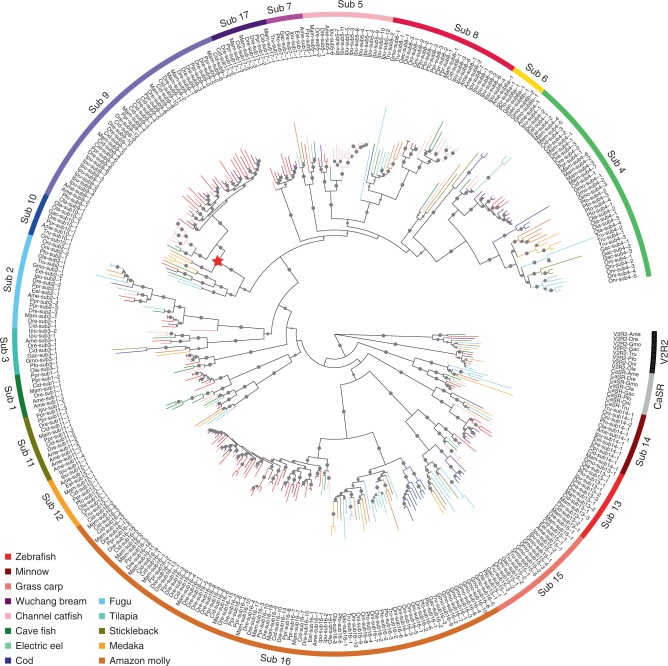
Evolutionary relationships of intact *OlfC* genes from 13 fish species. Tree was reconstructed using RAxML (version 8.1.17) and rooted with fish *CaSR* and *V2R2* genes. *OlfC* genes from different species are indicated by different colors. The gene expansion event specific to the ostariophysan fishes having the fright reaction is represented by a red star. Dre, *Danio rerio*, Zebrafish; Ppr, *Pimephales promelas*, Minnow; Cid, *Ctenopharyngodon idella*, Grass carp; Mam, *Megalobrama amblycephala*, Wuchang bream; Ipu, *Ictalurus punctatus*, Channel catfish; Ame, *Astyanax mexicanus*, Cave fish; Eel, *Electrophorus electricus*, Electric eel; Gmo, *Gadus morhua*, Cod; Tru, *Takifugu rubripes*, Fugu; Oni, *Oreochromis niloticus*, Tilapia; Gac, *Gasterosteus aculeatus*, Stickleback; Ola, *Oryzias latipes*, Medaka; Pfo, *Poecilia formosa*, Amazon molly. Detailed tree with species and gene names and bootstrap values is shown in Supplementary Fig. S3

In order to examine the pattern of expansion in the *OlfC* gene family and to evaluate whether gain or loss of gene members in specific subfamilies might be responsible for specific functions, we constructed a maximum likelihood (ML) tree of all the aligned *OlfC* genes (Fig. 2 and Supplementary Fig. 3). This tree showed that there are 16 or 17 subfamilies in teleost *OlfC* genes, which is generally consistent with previous studies^31,35,36^. Most subfamilies form monophyletic groups with high bootstrap support, except subfamily 6, which is divided into two groups. The phylogenetic tree also identified many species or lineage-specific gene duplications, such as those seen in subfamilies 4, 5, 8, 9 and 16, suggesting potential functional specialization. Intriguingly, among these expanded subfamilies, only subfamily 9 was expanded exclusively within ostariophysan fishes having the fright reaction, hinting that these genes may be candidates contributing to the unique fright reaction of ostariophysan fishes (Supplementary Fig. 3, Datas 2, 3).

**Fig. 3:**
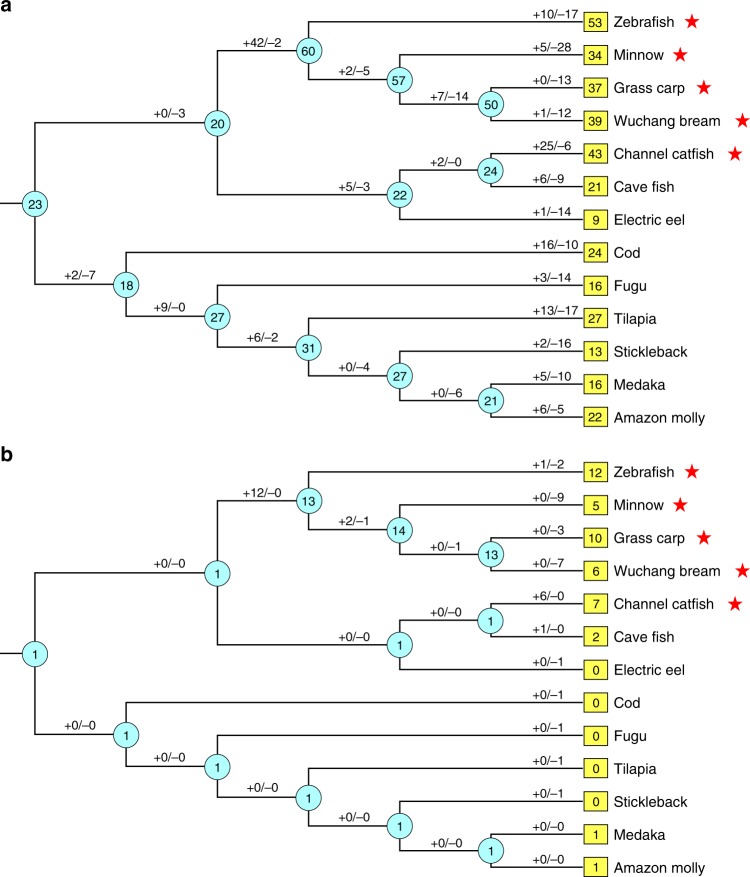
Evolutionary dynamics of intact *OlfC* genes in fishes. Both intact *OlfC* genes (**a**) and *OlfC* subfamily 9 (**b**) are examined. The estimated numbers of *OlfC* gene gains and losses are shown on each branch with plus and minus signs, respectively. The estimated number of genes for the ancestral nodes is shown in the cyan circles, and the number of intact *OlfC* genes in extant fish species is shown in the yellow boxes. Fish species having fright reaction are marked with red stars

To further characterize the evolutionary dynamics of *OlfC* gene repertoire size among fish species, we estimated the numbers of gene birth and death events and predicted the number of ancestral gene numbers based on comparison between the gene and species trees using a reconciliation analysis^37^. Our analyses showed that the ancestral numbers of *OlfC* genes were relatively stable across the evolution of *OlfC* genes, implying that the difference in *OlfC* gene numbers among extant fishes were mostly derived from recent lineage-specific expansions and contractions (Fig. 3a). For example, among the 9 extant fish species with more than 20 *OlfC* genes, 7 have massive lineage- or species-specific gene gains (*n* > 10). Similarly, recent losses (*n* > 10) were observed in all 4 extant fish species with <20 *OlfC* genes. It should be noted that the two highest gene gains (*n* = 42 and *n* = 25) were detected in the ancestral branch leading to zebrafish, minnow, grass carp, and Wuchang bream, and the terminal branch of channel catfish, all ostariophysan fishes having the fright reaction (Fig. 3a).

Considering the observation in the phylogenetic tree that the *OlfC* subfamily 9 expanded exclusively within ostariophysan fishes having fright reaction, we further examined gene birth and death events within *OlfC* subfamily 9 genes (Fig. 3b). Our results showed that *OlfC* subfamily 9 expanded independently in the ancestral branch for zebrafish, minnow, grass carp, and Wuchang bream, and in the terminal lineage leading to channel catfish. Considering that concerted evolution can generate a higher sequence similarity between paralogous genes than between orthologous genes^38^, we further test for gene conversion events among the *OlfC* subfamily 9 genes using Sawyer’s method, as implemented in the software GENECONV^39^. We indeed identified several possible events of gene conversion among *OlfC* subfamily 9 genes in channel catfish (Supplementary Data 4 and Table 2), suggesting that concerted evolution may result in the independent expansion of *OlfC* subfamily 9 genes in the ancestral branch of zebrafish, minnow, grass carp, and Wuchang bream, and in the terminal lineage leading to channel catfish. Taken together, all these analyses indicate that *OlfC* subfamily 9 expanded in ostariophysan fishes having the fright reaction, which supports the hypothesis that genes within *OlfC* subfamily 9 have an important role in the fright reaction in ostariophysan fishes.

### Association of *OlfC* subfamily 9 with the fright reaction

**Fig. 4:**
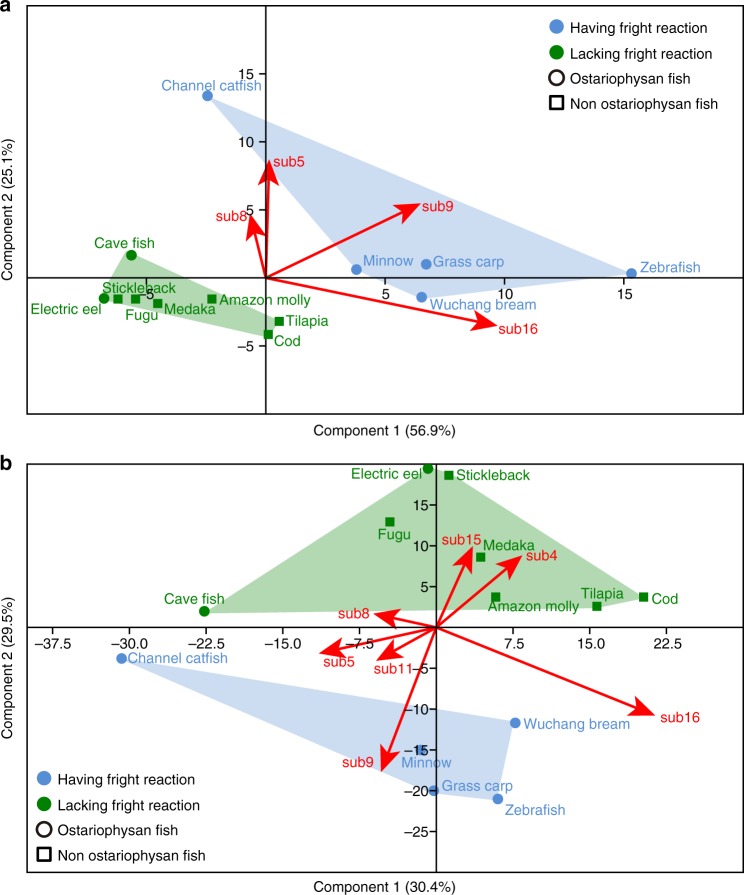
Scatterplots showing the results of the principal component analysis (PCA) analysis of intact *OlfC* genes with their respective *OlfC* gene subfamilies. Both *OlfC* gene counts (**a**) and proportion (**b**) across each subfamily are shown. First and second axes explain more than 82% and 59.9% of the variance within the data set. Blue polygons represent fishes having fright reaction and green polygons represent fishes lacking fright reaction. Red arrows represent the contribution of particular subfamilies on the positioning of each species on the plots

We used principal component analysis (PCA) to identify and visualize differences in the *OlfC* gene repertoires among ostariophysan fishes having and lacking the fright reaction and in non-ostariophysan fishes to identify *OlfC* subfamilies that might be these differences (Fig. 4). Our PCA results showed that fishes having the fright reaction grouped away from fishes without the fright reaction, whereas no separation was found between ostariophysan fishes and non-ostariophysan fishes (Fig. 4a and Supplementary Data 2). These results suggest that having a fright reaction or not played a role in determining the configuration of the fish *OlfC* gene subgenomes. The first two principal components (PCs) explained more than 82% variance of the fish *OlfC* gene repertoire size. An analysis of similarity (ANOSIM) showed that the fish *OlfC* gene repertoires varied significantly between the fishes having fright reaction and fishes lacking fright reaction (ANOSIM *R* = 0.73, *P* = 0.002), demonstrating that the repertoires of *OlfC* genes in each subfamily were correlated with fright reaction in ostariophysan fishes. The PCA analysis can also distinguish which *OlfC* gene subfamilies were driving the differences in *OlfC* gene repertoire among species. We found that increased size of *OlfC* subfamily 9 was most closely associated with fishes having fright reaction. These results are consistent both using gene count (Fig. 4a and Supplementary Data 2) and proportion (Fig. 4b and Supplementary Data 3) of the *OlfC* gene subfamilies (ANOSIM *R* = 0.73, *P* = 0.002, and ANOSIM *R* = 0.33, *P* = 0.02, respectively).

To further investigate whether fright reaction is associated with the number of *OlfC* genes while statistically controlling for phylogeny, we performed a phylogenetic logistic regression analysis, which is for binary variables^40^. We selected the trait “presence of a fright reaction” as the predictor variable for this analysis. According to the results from phylogenetic logistic regression analysis, having a fright reaction showed significant correlation with the number of functional *OlfC* genes (AIC = 8.59, *P* = 0.04). Taken together, all these analyses demonstrated that the presence of a fright reaction is an important factor that is associated with the number of functional *OlfC* genes.

### Profound changes in expression of *OlfC* genes after fright reaction

**Fig. 5:**
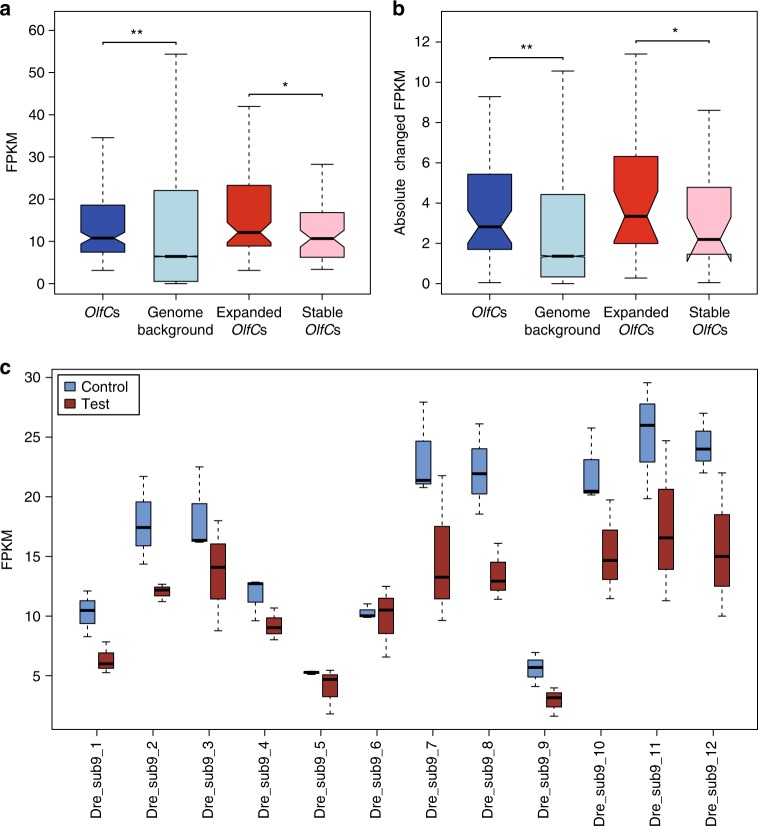
Comparison of the expression patterns of *OlfC* genes. **a** Expression levels, shown as fragments per kilobase per million (FPKM), were compared between all *OlfC* genes and non-*OlfC* genes, as well as expanded *OlfC* genes and non-expanded *OlfC* genes. **b** Absolute change of expression levels before and after fright reaction in the zebrafish were compared between all *OlfC* genes and non-*OlfC* genes, as well as expanded *OlfC* genes and non-expanded *OlfC* genes. **c** Mean FPKM values of zebrafish *OlfC* subfamily 9 before and after fright reaction. Significant differences are represented by asterisks, based on Wilcoxon’s rank-sum tests, **P < 0.001; *P *<* 0.05

Previous studies have shown that odor stimuli in mouse could decrease the transcription levels of OR genes in activated olfactory sensory neurons^41^, which may represent a quick adaptation of sensory neurons to a continual stimulus^42^. Therefore, looking for genes whose expression decreases after fright reaction could reveal the receptors for the alarm signal. Since fishes do not have vomeronasal organs, most *OlfC* genes are expressed in the olfactory epithelium of the nasal cavity^43^. Therefore, we examined the expression patterns of *OlfC* genes in olfactory epithelium transcriptomes before and after fright reaction in zebrafish using RNA-sequencing (RNA-seq) with triplicates samples (Supplementary Table 3, Data 5, and Fig. 4a). The expression levels of *OlfC* genes were statistically significantly increased above the expression level of all the other genes across the genome in the normal control condition (Wilcoxon’s rank-sum test, *P* < 0.001) (Fig. 5a), confirming that the olfactory epithelium is a tissue where *OlfC* genes are expressed. Interestingly, our results also showed that the expression levels of the expanded *OlfC* genes were significantly higher than for genes in subfamilies that had not expanded (Wilcoxon’s rank-sum test, *P* = 0.019) (Fig. 5a). These results suggested that genes in the expanded *OlfC* genes might have more important functional roles than those in the subfamilies that had not expanded in detecting water-soluble pheromones in zebrafish.

As the fright reaction should have substantial impact on behavioral and physiological changes in zebrafish, we hypothesized that they might be reflected by changes in gene expression. Consistent with this hypothesis, we found that the absolute difference in the expression levels of *OlfC* genes before and after the fright reaction in zebrafish were significantly higher than those for the other genes in the genome (Wilcoxon’s rank-sum test, *P* < 0.001) (Fig. 5b and Supplementary Fig. 4B). Similarly, our results also exhibited much more profound changes for expression of the expanded *OlfC* genes than for the non-expanded *OlfC* genes before and after the fright reaction (Wilcoxon’s rank-sum test, *P* *<* 0.05) (Fig. 5b). Furthermore, our results also demonstrated that the transcription levels of expanded *OlfC* subfamily 9 genes decreased after fright reaction (Fig. 5c), suggesting that these genes may be the receptors of the odors for fright reaction. Taken together, these analyses indicate that *OlfC* genes, especially the expanded *OlfC* genes, might play an important role in the fright reaction in zebrafish.

### Expanded *OlfC* subfamily genes are subjected to positive selection

**Fig. 6:**
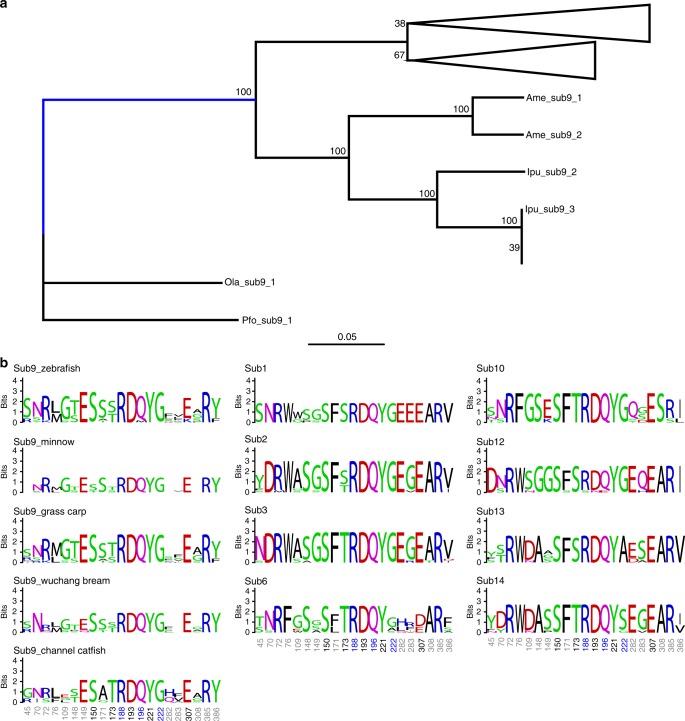
Selective analysis of OlfC. **a** ML gene tree for *OlfC* subfamily 9 from all fishes. Branch with blue color indicates the foreground branch in branch-site model analysis. Numbers on the tree are bootstraps for each node with branches collapsed for low bootstrap support (<70). **b** Sequence logos for the predicted binding pocket residues in OlfC. Proximal sites predicted to be important for direct ligand “binding” are marked in black, distal binding sites responsible for binding “selectivity” are marked in gray, and the sites thought to be for structural maintenance are shown in blue. Note that the sites responsible for binding “selectivity” (marked in gray) showed much more variability in expanded OlfC subfamily 9 in ostariophysan fishes compared with other non-expanded OlfC subfamilies

Previous studies have shown that positive selection serves as a major driving force for the expansion of gene families for particular functional roles^44^. To better understand the evolutionary dynamics of expanded *OlfC* subfamily 9 genes associated with flight reaction in ostariophysan fishes, we analyzed the selective pressure acting on these genes using PAML (Table 1 and Supplementary Data 6). As expected, the site model analyses showed that the selection model (M8) fitted significantly better than neutral models (i.e., M7 and M8a; Table 1, *P* = 1.0 × 10^−10^ and *P* = 1.7 × 10^−4^, respectively), indicating that functional diversification and adaptation has occurred in the expanded members of *OlfC* subfamily 9 genes in ostariophysan fishes having the fright reaction. To test whether the evidence for positive selection was restricted to the ancestral branch, the branch-site model was further employed. We found that the ancestral branch of *OlfC* subfamily 9 genes of ostariophysan fishes was significantly driven by positive selection (*P* = 0.02, Table 1 and Fig. 6a). Sites showing evidence of positive selection were mapped to the predicted secondary structure of zebrafish OlfC subfamily 9 protein sequences (Supplementary Fig. 5). We found that the majority of the positively selected sites were located in the N-terminal extracellular region, which is consistent with a previous study in rodents^45^. As the N-terminal extracellular region is thought to be the ligand-binding domain^23,46,47^, this result suggested that positive selection has important roles in driving changes in the binding capability of members of the expanded *OlfC* subfamily 9 genes in ostariophysan fishes.

**Table 1:** The parameters and statistical significances of likelihood ratio tests in the *OlfC* subfamily 9

Models	ln *L*^a^	np^b^	Models compared	2Δln *L*^c^	d.f.	*P* value^d^
Site models						
A: M1a (nearly neutral)	−36,829.61	82	B vs. A	0	2	1
B: M2a (positive selection)	−36,829.61	84				
C: M7 (beta)	−36,515.43	82	D vs. C	75.04	2	**1.0** × **10**^**–10**^
D: M8 (beta and *ω*)	−36,477.91	84				
E: M8a (beta and *ω*_s_ = 1)	−36,484.98	83	D vs. E	14.14	1	**1.7** × **10**^**–4**^
Branch-site model						
F: Model A	−36,826.55	84				
G: Null model A (*ω*_2_ = 1)	−36,829.23	83	F vs. G	5.36	1	**0.02**

^a^The natural logarithm of the likelihood value

^b^Number of parameters

^c^Twice the difference in ln *L* between the two models compared

^d^*P* values lower than 0.05 are shown in bold

Site-directed mutagenesis has found that changes in the amino acid sequence near in the N-terminal extracellular domain of *OlfC* genes have different functional effects on ligand binding^28,48^. For example, proximal sites are thought to be important for direct ligand “binding,” while distal binding sites are thought to be crucial for binding “selectivity” and structural sites were involved in structural interaction^48^. To test whether expansion of *OlfC* subfamily 9 in ostariophysan fishes with a fright reaction contributed to the functional diversification of their ligand binding, we generated sequence logos for these sequences to compare sequence conservation between *OlfC* subfamily 9 genes in ostariophysan fishes and the sequences from other subfamilies that did not increase subfamily size (Fig. 6b). Our results clearly show that amino acid sites essential for functions in OlfC are highly conserved, both within subfamilies that have and have not expanded in size. However, differences in the degree of sequence conservation at sites responsible for binding “selectivity” are observed. That is, OlfC subfamily 9 sequences showed much more variability in the expanded OlfC subfamily 9 in each species in ostariophysan fishes compared to other non-expanded OlfC sequences (Fig. 6b, left column vs. middle column and right column). This analysis suggested that expansion of *OlfC* subfamily 9 genes in ostariophysan fishes having fright reaction led to changes in ligand binding “selectivity,” which might contribute to their ability to detect various waterborne pheromones.

## Discussion

Elucidating evolutionary mechanisms and selective forces shaping complex traits across the tree of life is an ultimate goal in evolutionary biology^49^. As the largest group of freshwater fishes, ostariophysans likely enter freshwater around 251 million years ago. This pioneering exploration was hypothesized to be driven by the global decrease in oxygen levels in seawater caused by the largest mass extinction at the end of the Permian era^4^. However, the first pioneers must have faced a more challenging habitat, due to the different types of predators and a smaller water area. Fortunately, most ostariophysans evolved a pheromone-mediated fright reaction to defend against predators, which is thought to contribute to their successful diversification^11^. Ostariophysan fishes form a monophyletic group, which included five major groups: Gonorynchiformes (milkfishes and sandfishes), Cypriniformes (minnows, loaches, and carps), Characiformes (tetras and their allies), Siluriformes (catfishes), and Gymnotiformes (electric eels)^2,4,5,50,51^. Some members of ostariophysan fishes do not possess a fright reaction, such as blind cave fishes, armored catfishes, and electric fishes^11^. Interestingly, all the ostariophysan fishes lacking the fright reaction seem to be either cave dwelling, armored, or electric, suggesting that they have evolved specific mechanisms to adapt to the freshwater environment, and do not need the fright reaction. Together, this evidence supports a hypothesis that ostariophysan fishes having a fright reaction have evolved a specific sense to detect the pheromones mediating the fright reaction^11^.

In accordance with above hypothesis, we found that ostariophysan fishes having a fright reaction have a larger repertoire of *OlfC* genes compared to ostariophysan fishes without a fright reaction and to non-ostariophysan fishes. Although it remains unclear which aspects of the pheromone detection ability the numbers of *OlfC* genes reflects, it is reasonable to conclude that species with higher numbers of *OlfC* genes have an ability to detect an expanded range of substrates. Therefore, our findings represent a perfect example of the correlation between evolutionary patterns with function for a gene family. We further found that subfamily 9 of the *OlfC* had specifically been expanded in ostariophysan fishes having a fright reaction. We assume that the exclusive expansion of *OlfC* subfamily 9 genes in ostariophysan fishes might be attributable to their fright reaction ability. Similarly, the species-rich cichlids (which are not ostariophysan fish) also expanded their *OlfC* gene repertoire to detect a variety of amino acids, which is thought to have contributed to their extraordinary diversification^31^. However, among *OlfC* subfamilies, subfamily 9 was not expanded in the cichlids. These observations further support the conclusion that the expansion of subfamily 9 of *OlfC* genes in ostariophysan fishes might have had an important role in the evolution of the fright reaction.

Although there is little direct experimental evidence addressing the function of the expanded *OlfC* gene family in ostariophysan fishes having fright reaction, the pattern of evolution and expression strongly indicate an important role of these *OlfC* genes in the fright reaction: (1) ostariophysan fishes possess a fright reaction elicited by pheromones, (2) fish *OlfC* genes detect pheromones dissolved in water, (3) some ostariophysan fishes lack the fright reaction, but are either cave dwelling, armored, or electric, (4) *OlfC* genes were specifically expanded in ostariophysan fishes having a fright reaction, but not in ostariophysan fishes without fright reaction or non-ostariophysan fishes, (5) expression levels of *OlfC* genes in subfamilies that have increased in number change significantly, compared to *OlfC* genes in non-expanded subfamilies, after the fright reaction in zebrafish, and (6) the ligand-binding region of OlfC proteins in the expanded subfamily show evidence of positive selection, especially within the ligand binding “selectivity” region that has been experimentally defined in zebrafish. Together, these lines of evidence indicate that expanded *OlfC* genes in ostariophysan fishes might have contributed to the fright reaction.

Previous studies have found that the evolution of chemosensory receptor genes is largely driven by genomic drift and is independent of selection^30,52^. In contrast, comparative genomic analyses have found that the evolution of OR repertoires have in part been shaped by natural selection, and reflects ecological adaptations^53–56^. Consistent with this, our results further provide new evidence that natural selection has a role in shaping the evolution of the *OlfC* gene repertoires. We found that the independent expansion of *OlfC* subfamily 9 is associated with the fright reaction in ostariophysan fishes. We speculate that, given a link between *OlfC* subfamily 9 and the fright reaction in ostariophysan fishes, these *OlfC* genes might be directly involved in the fright reaction. Further functional studies will be needed to confirm this hypothesis.

## Methods

### Data sources

Genome sequences from a total of 13 fish species, including 5 ostariophysan fishes having fright reaction [zebrafish (*Danio rerio*), minnow (*Pimephales promelas*), grass carp (*Ctenopharyngodon idella*), Wuchang bream (*Megalobrama amblycephala*), and channel catfish (*Ictalurus punctatus*)], 2 ostariophysan fishes lacking fright reaction [cave fish (*Astyanax mexicanus*) and electric eel (*Electrophorus electricus*)], and 6 non-ostariophysan fishes [cod (*Gadus morhua*), fugu (*Takifugu rubripes*), tilapia (*Oreochromis niloticus*), stickleback (*Gasterosteus aculeatus*), medaka (*Oryzias latipes*), and Amazon molly (*Poecilia formosa*)], were used in this study. Genome sequences, except minnow, grass carp, Wuchang bream, channel catfish, and electric eel, were downloaded from Ensembl version 83 (http://www.ensembl.org)^57^. The genome sequence of minnow was obtained from https://www.setac.org/page/fhmgenome^58^. The genome sequence of grass carp was obtained from http://www.ncgr.ac.cn/grasscarp/^59^. The genome sequence of Wuchang bream was obtained from http://gigadb.org/dataset/view/id/100305^60^. The genome sequence of channel catfish was obtained from http://www.ncbi.nlm.nih.gov/^61^. The genome sequence of electric eel was obtained from http://efishgenomics.zoology.msu.edu/^62^.

### Gene identification

To identify *OlfC* genes in each of the 13 fish genomes, we followed an earlier study^35^ with minor modifications. First, we used previously published V2R protein sequences from vertebrates^34^ as queries to conduct TBLASTN^63^ searches against each of the 13 fish genome sequences, with an *e* value cutoff of 1 × 10^−10^. Second, redundant sequences that hit on the same genomic regions were filtered and sequences shorter than 200 nucleotides were discarded. Third, genomic sequences of homologous genes were extended in the 5′ and 3′ directions and protein-to-genomic sequence alignment with the known V2R protein sequences was conducted using GeneWise^64^. Finally, the protein sequences of identified putative *OlfC* genes were used in BLASTP against the NR database to ensure that the best hit was a *V2R* gene. We classified the identified *OlfC* genes into three categories: intact genes (I), truncated genes (T), and pseudogenes (P). Intact genes were defined as genes with an intact ORF and complete coding region. Truncated genes were defined as genes with an intact ORF but partial coding region. Pseudogenes were defined as genes with a disruptive ORF because of nonsense or frameshift mutations in the coding region.

### Evolutionary analysis

A total of 354 intact *OlfC* genes were analyzed with *CaSR* and *V2R2* genes used as outgroups^31^. All coding sequences from the *OlfC*, *CaSR*, and *V2R2* genes were translated into protein sequences and subsequently aligned with the program MUSCLE^65^. Phylogenetic analyses were conducted using both ML and neighbor-joining (NJ) approaches. The ML tree was constructed by RAxML (version 8.1.17)^66^ under a JTT + G substitution model with bootstrap support values determined using 1000 replicates. The NJ tree was reconstructed using the Poisson protein distances^67^ and pairwise deletion of gap sites implemented in MEGA5^68^ and was evaluated with 1000 replicates using the bootstrap method^69^.

To estimate gains and losses of *OlfC* genes across the fish phylogeny, the reconciled-tree method implemented in the program NOTUNG 2.6^37^ was carried out by comparing the gene tree with the species tree. The gene tree topology was taken from our ML tree, while the species tree topology was taken from recent studies^2,4,50^. Gene gains and losses were inferred across each branch of the species tree and ancestral nodes using the incongruence between the gene and species trees and the parsimony principle.

### Detection of gene conversion events

Sequence alignments were generated as described above in evolutionary analysis. The program GENECONV^70^ was used to identify gene conversion events, which employs permutation to detect whether gene conversion tracts are statistically significant given the distribution of mismatches in the entire sequence alignment.

### PCA and analysis of similarities

PCA was conducted using the program PAST v2.17c^71^ on all intact *OlfC* genes to explore the degree of correlation between specific *OlfC* gene subfamilies with the fright reaction in ostariophysan fishes. The PCA algorithm used was the matrix of the *OlfC* gene data, which was then employed to assess patterns of variation in *OlfC* gene subfamily distribution between fishes having the fright reaction and fishes lacking the fright reaction. Both the number and the proportion of each *OlfC* subfamily were analyzed. The statistically significant difference between the above two groupings was examined using a nonparametric test for ANOSIM^72^ based on the Euclidean distances among all observations.

### Phylogenetic logistic regression analysis

We used phylogenetic logistic regression analysis, which is for binary variables^40,73^, to investigate the relationship between the number of functional *OlfC* gene and the trait of whether having fright reaction while statistically controlling for phylogeny. We performed this analysis using R with the phylolm package (https://cran.r-project.org/web/packages/phylolm/index.html). The phylogeny of these fishes was constructed from *cytb* genes from these species. The trait of whether having fright reaction was coded each as 1 (having fright reaction) and 0 (lacking fright reaction).

### RNA-seq data analysis

The ethics committee of the Institute of Hydrobiology, Chinese Academy of Sciences, approved all animal experiments. Two groups of adult wild-type zebrafish with the AB background were maintained in the zebrafish facility for 1 week to familiarize with the laboratory environment. One group was used as the control group, and the other group was used as a test group. In the test group, we made shallow lesions on the skin of an adult zebrafish (5 to 6 on each side) using a sharp razor, immersed the fish and washed the damaged skin with distilled water, and then dropped the water into the tank using syringe with a long tube similar to a previous study^74^. When the test group zebrafish showed a severe fright reaction after the alarm substances were introduced in about 10 min, they were anesthetized immediately and their olfactory mucosae dissected from them at 4 °C. Olfactory mucosae from the control group zebrafish were also collected. Olfactory mucosae were immediately frozen in liquid nitrogen. Tissues from three animals were pooled to obtain sufficient RNA for analysis. Three independent biological replicates for both the control and test groups were prepared. RNA isolation, RNA-seq library construction, and sequencing were conducted by Novogene (Beijing, China) following the approach of our previous study^75^. Raw reads were assessed using FastQC (https://www.bioinformatics.babraham.ac.uk/projects/fastqc/) and filtered using Trim galore (version 0.3.7) (http://www.bioinformatics.babraham.ac.uk/projects/trim_galore/) to remove any adaptor sequence and to trim any bases with Phred quality scores lower than 20. Only paired-end reads where both reads were longer than 50 bp after trimming were retained for the subsequent analysis. High-quality paired-end reads from each sample were separately aligned to the transcript sequences for zebrafish from Ensembl (release 83)^76^ using Bowtie (version 1.1.1)^77^, and transcript abundances were estimated using RSEM program (v1.2.20)^78^. Gene expression levels (fragments per kilobase per million (FPKM)) were calculated by RSEM and only genes with FPKM >1 in at least half of the samples were considered as transcriptionally active genes and used in the subsequent analysis. Raw read counts for each gene detected from RSEM were extracted and normalized to control for differences in sequencing depth using TMM method and differentially expressed genes were identified using the edgeR package^79^ using a minimal fold change of 2 and an adjusted *P* value cutoff of 0.05.

### Positive selection analysis

Coding sequences of *OlfC* subfamily 9 that were predicted to have expanded only in ostariophysan fishes having the fright reaction were translated into their protein sequences, aligned with MUSCLE^65^, and then back-translated into nucleotide-coding sequences. *OlfC* subfamily 9 gene tree was constructed using ML methods. To detect signatures of positive selection, we employed site model and branch-site model in the codeml program (PAML 4.7 package)^80^ using ML gene tree as input. Specially, three pairs of paired site models were tested: M1a (nearly neutral: *ω*_0_ < 1, *ω*_1_ = 1) vs. M2a (positive selection: *ω*_0_ < 1, *ω*_1_ = 1, *ω*_2_ > 1), M7 (nearly neutral; beta distribution: 0 < *ω*_0_ < 1) vs. M8 (*p* positive selection; beta distribution: 0 < *ω*_0_ < 1 and *ω*_1_ > 1), and M8a (nearly neutral; beta distribution: 0 < *ω*_0_ < 1 and *ω*_1_ = 1) vs. M8 (positive selection; beta distribution: 0 < *ω*_0_ < 1 and *ω*_1_ > 1). In the branch site model (model = 2, Nsites = 2), the neutral model constrains a class of sites to have *ω* = 1 (fix_omega = 1, omega = 1), and the selection model allows a class of codons on the foreground branch to have *ω* > 1 (fix_omega = 0, omega = 1.5). Likelihood ratio tests were used to compare these nested models, where twice the log likelihood value differences with a *χ*^2^ distribution was used to identify positively selected codons. The sites under positive selection were detected by the Bayes empirical Bayes method^81^. Because we are focusing on the specific expanded *OlfC* subfamily 9, all these positive selection analyses were using *OlfC* subfamily 9 gene sequences after removing genes showing evidence of gene conversion. Sequence conservation was visualized using WebLogo^82^ for functional residues in OlfC proteins for the expanded OlfC subfamily 9 in each species from ostariophysan fishes, and for other non-expanded OlfC subfamilies in all fishes species. The protein membrane topology for OlfC subfamily 9 proteins was created using Protter^83^, and the locations of putative positively selected sites detected by different methods were marked by star with different colors.

### Reporting summary

Further information on research design is available in the Nature Research Reporting Summary linked to this article.

## Supplementary information

Description of additional supplementary items

Supplementary Information

Reporting Summary

Supplementary Data 1

Supplementary Data 2

Supplementary Data 3

Supplementary Data 4

Supplementary Data 5

Supplementary Data 6

## Data Availability

The sequencing data have been deposited into the National Center for Biotechnology Information (NCBI) Sequence Read Archive (SRA) database (accession no. SRP154651).

## Code availability

The custom scripts have been deposited in Github (https://github.com/yangliandong/OlfC_evolution).
