# Supplementary material for: Expansion of vomeronasal receptor genes (OlfC) in the evolution of fright reaction in Ostariophysan fishes
Source: Commun Biol. 2019 Jun 21;2:235. doi: 10.1038/s42003-019-0479-2 (PMC6588630; doi:10.1038/s42003-019-0479-2)
Supplement: Supplementary file 1 — Description of additional supplementary items [file 42003_2019_479_MOESM1_ESM.docx]

**Description of Additional Supplementary items**

**File Name**: Supplementary Data 1

**Description**: Amino acid sequences of the OlfC genes identified from 14 fish species (fasta format).

**File Name**: Supplementary Data 2

**Description**: Number of *OlfC* gene in different subfamilies across fish genomes (excel file).

**File Name**: Supplementary Data 3

**Description**: Proportion (%) of *OlfC* gene in different subfamilies across fish genomes (excel file).

**File Name**: Supplementary Data 4

**Description**: Amino acid sequences of the *OlfC* genes used for GENECONV analysis (fasta format).

**File Name**: Supplementary Data 5

**Description**: Data matrix of per-sample and per-gene read counts calculated by RSEM (excel file).

**File Name**: Supplementary Data 6

**Description**: Coding sequences of the *OlfC* genes used for positive selection analysis(fasta format).
