# Supplementary material for: Expansion of vomeronasal receptor genes (OlfC) in the evolution of fright reaction in Ostariophysan fishes
Source: Commun Biol. 2019 Jun 21;2:235. doi: 10.1038/s42003-019-0479-2 (PMC6588630; doi:10.1038/s42003-019-0479-2)
Supplement: Supplementary file 2 — Supplementary Information [file 42003_2019_479_MOESM2_ESM.pdf]

Supplementary Information for

Expansion of vomeronasal receptor genes (*OlfC*) in the evolution of fright reaction in Ostariophysan fishes

Table of contents

Supplementary Figures
Supplementary Tables

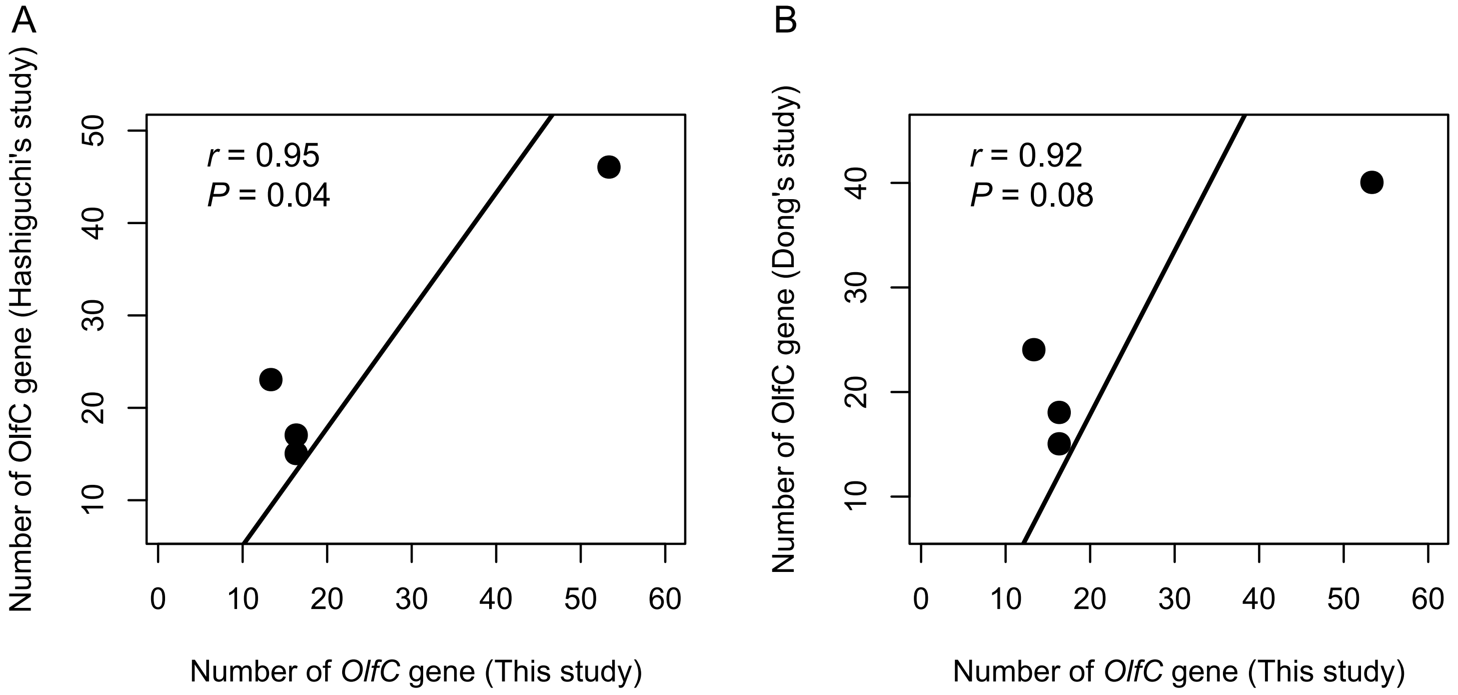

Supplementary Figure 1 | Comparison of *OlfC* gene counts between our study with previous reports.

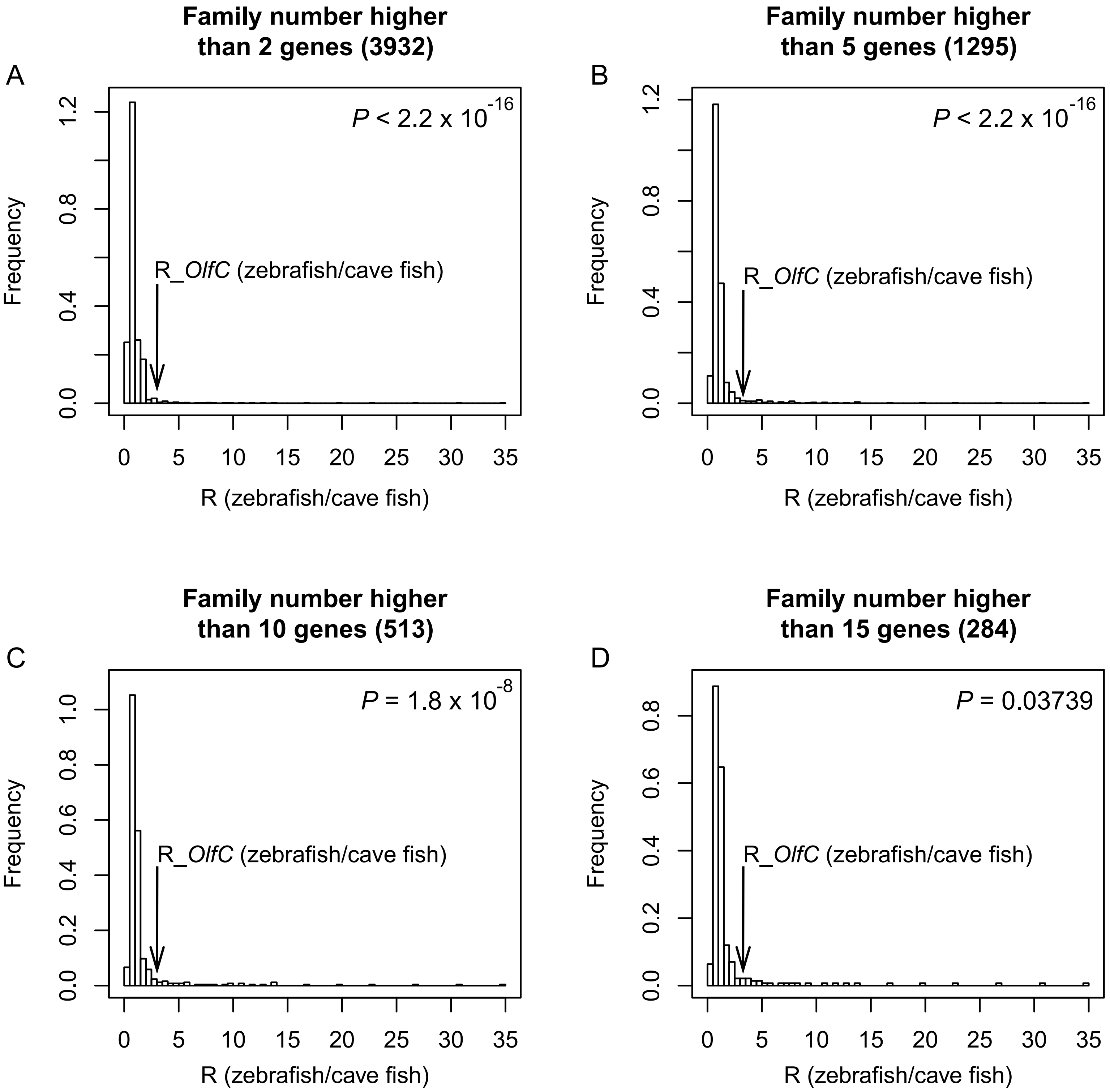

Supplementary Figure 2 | Frequency distribution of the ratio (R) between zebrafish and cave fish for the relative size of gene families from their genomes. Arrow shows the R value for *OlfC* gene.

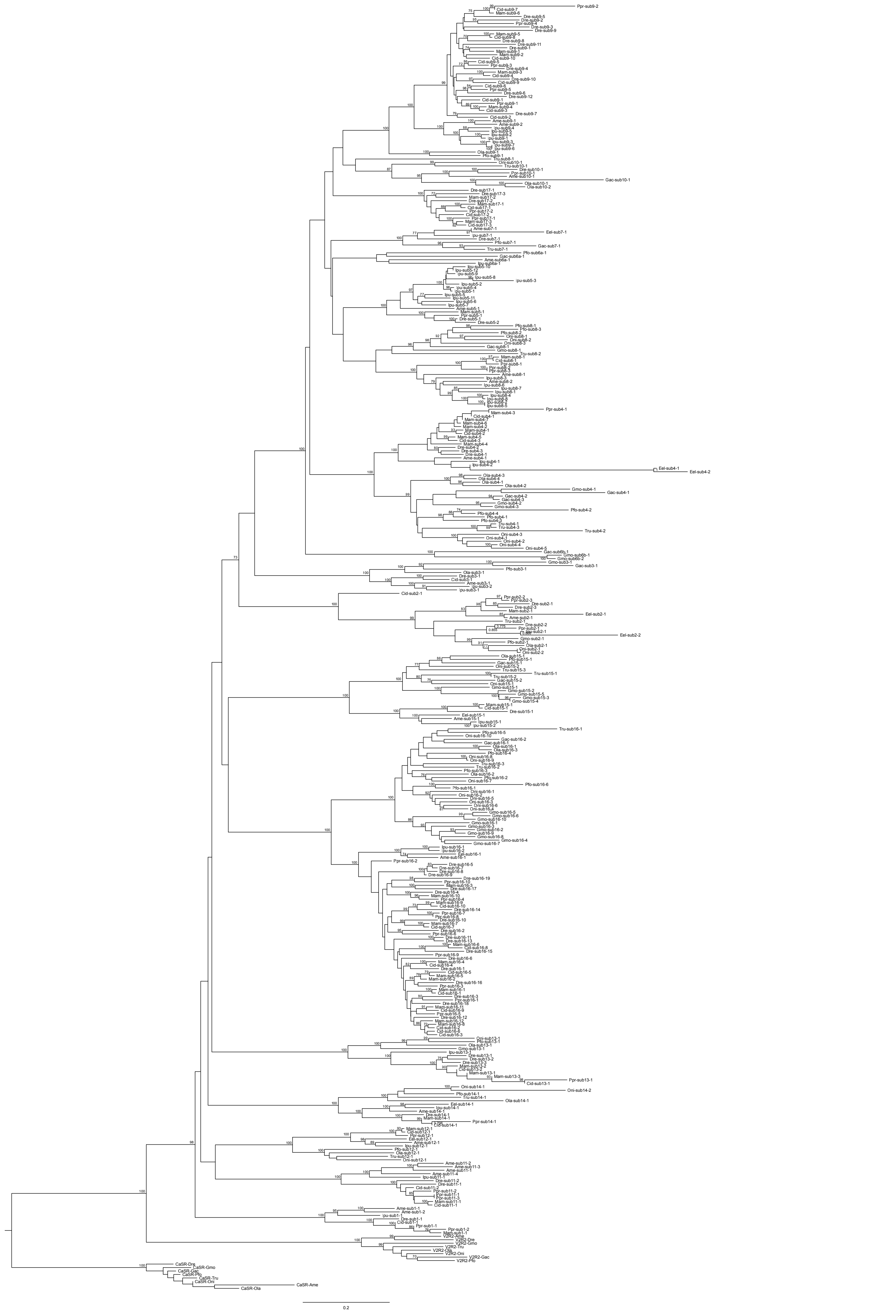

Supplementary Figure 3 | The Neighbor-Joining (NJ) phylogenetic tree with detailed species and gene names. Bootstraps were shown. Bootstrap values higher than 70 were shown.

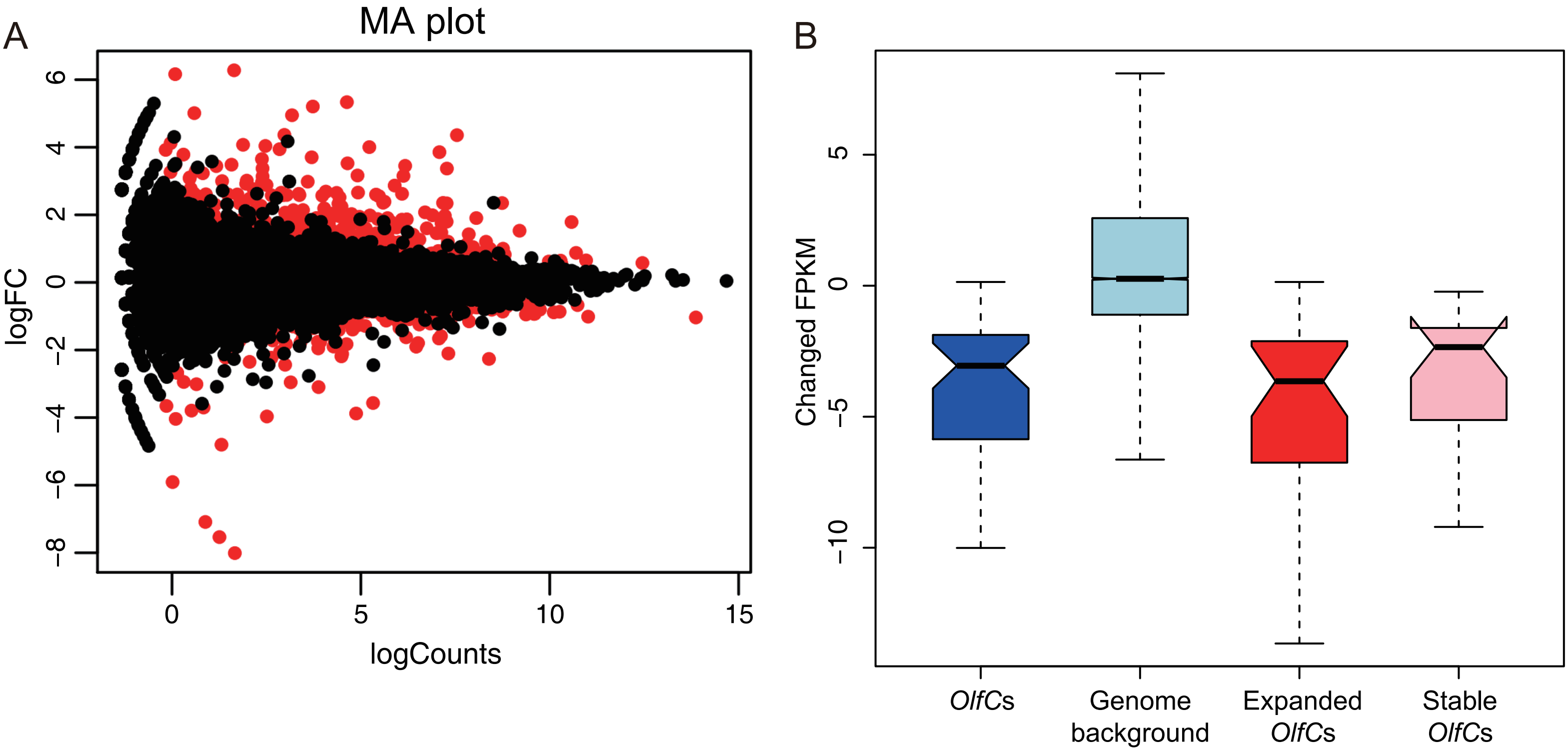

Supplementary Figure 4 | Expression analyses between test and control groups of zebrafish after fright reaction. (A) The MA plot of all genes in zebrafish between test and control groups with red color for differentially expressed genes. (B) Change of expression levels before and after fright reaction.

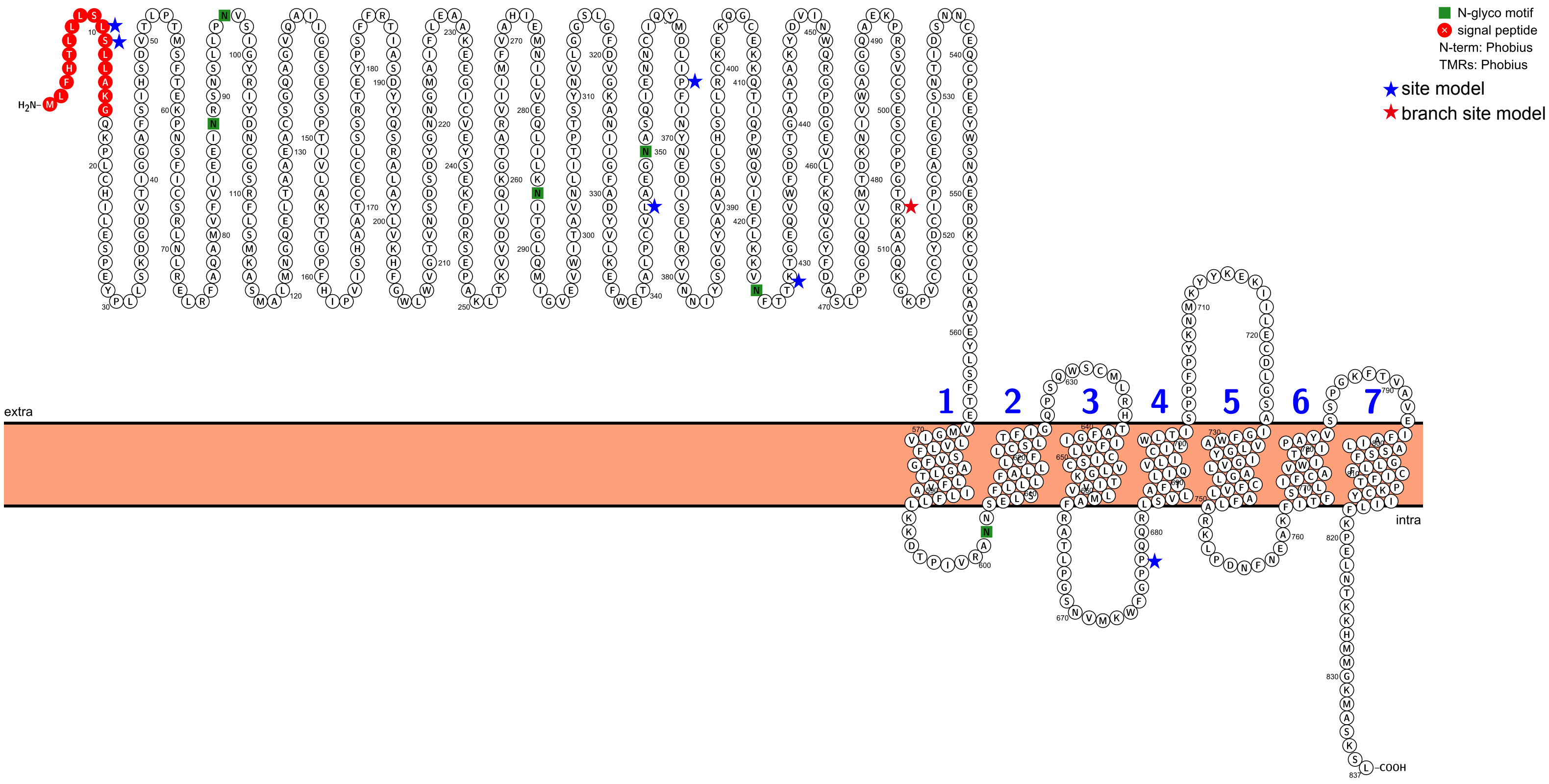

Supplementary Figure 5 | Structure domains in OlfC. Membrane topology model generated by Protter for OlfC subfamily 9 protein, and the locations of putative positively selected sites detected by different methods were marked by star with different colors.

Supplementary Table 1 | Comparison of *OlfC* gene counts between our study with previous reports.

Species	Number of OlfC gene (This study)	Number of OlfC gene (Hashiguchi's study) [1]	Number of OlfC gene (Dong's study) [2]
Zebrafish	53	46	40
Medaka	16	17	15
Stickleback	13	23	24
Fugu	16	15	18

Supplementary Table 2 | Potential gene conversion events between Channel catfish *OlfC* subfamily 9 genes.

Sequence pair	P value	BC KA P value	Fragment length
Ipu_sub9_1 and Ipu_sub9_5	0.0002	0.00119	136 aa
Ipu_sub9_4 and Ipu_sub9_5	0.0003	0.00212	186 aa

Supplementary Table 3 | Summary of the RNA-seq datasets in zebrafish olfactory mucosae transcriptome before and after fright reaction.

Tissue	Sample accession	SRA accession	Status	Read number	% Mapped
olfactory mucosae	OE_c1	SRR7549094	Control 1	26,055,310	67.6
olfactory mucosae	OE_c2	SRR7549095	Control 2	22,776,726	66.2
olfactory mucosae	OE_c3	SRR7549096	Control 3	22,428,587	67.7
olfactory mucosae	OE_t1	SRR7549097	Test 1	25,131,021	70.6
olfactory mucosae	OE_t2	SRR7549092	Test 2	21,307,584	64.7
olfactory mucosae	OE_t3	SRR7549093	Test 3	26,578,173	67.0

References

1 Hashiguchi, Y., Furuta, Y. & Nishida, M. Evolutionary patterns and selective pressures of odorant/pheromone receptor gene families in teleost fishes. *Plos One* **3**, e4083, doi:10.1371/journal.pone.0004083 (2008).

2 Dong, D., Jin, K., Wu, X. & Zhong, Y. CRDB: database of chemosensory receptor gene families in vertebrate. *Plos One* **7**, e31540, doi:10.1371/journal.pone.0031540 (2012).
